# Effects of intraoperative diltiazem infusion on flow changes in arterial and venous grafts in coronary artery bypass graft surgery

**DOI:** 10.5935/1678-9741.20150045

**Published:** 2015

**Authors:** Ozan Erdem, Mehmet Erdem Memetoğlu, Ali İhsan Tekin, Ümit Arslan, Özgür Akkaya, Rasim Kutlu, İlhan Gölbaşı

**Affiliations:** 1 Akdeniz University School of Medicine, Antalya, Turkey.; 2 Denizli State Hospital, Denizli, Turkey.

**Keywords:** Flow Measurements, Coronary Artery Bypass, Diltiazem, Myocardial Revascularization

## Abstract

**Objective:**

This study aimed to show the effects of intra-operative diltiazem infusion on flow in arterial and venous grafts in coronary artery bypass graft surgery.

**Methods:**

Hundred fourty patients with a total of 361 grafts [205 (57%) arterial and 156 (43%) venous] underwent isolated coronary surgery. All the grafts were measured by intraoperative transit time flow meter intra-operatively. Group A (n=70) consisted of patients who received diltiazem infusion (dose of 2.5 microgram/kg/min), and Group B (n=70) didn't receive diltiazem infusion.

**Results:**

Mean graft flow values of left internal mammary artery were 53 ml/min in Group A and 40 ml/min in Group B (*P*<0.001). Pulsatility index (PI) values of left internal mammary artery for Group A and Group B were 2.6 and 3.0 respectively (*P*<0.001). No statistically significant difference was found between venous graft parameters.

**Conclusion:**

We recommend an effect of diltiazem infusion in increasing graft flows in coronary artery bypass graft operations.

## Abstract (pt)

**Objetivo:**

Este estudo teve como objetivo mostrar os efeitos da infusão de diltiazem intraoperatória no fluxo arterial e enxertos venosos em cirurgia de revascularização do miocárdio.

**Métodos:**

Cento e quarenta pacientes com um total de 361 enxertos [205 (57%) arteriais e 156 (43%) venosos] passaram por uma cirurgia coronária isolada. Todos os enxertos foram medidos pelo medidor de fluxo de tempo de trânsito intraoperatório. Grupo A (n=70), formado por pacientes que receberam infusão de diltiazem (dose de 2,5 micrograma/kg/min), e Grupo B (n=70), por aqueles que não receberam infusão de diltiazem.

**Resultados:**

Os valores médios de fluxo de enxerto de artéria mamária interna esquerda foram 53 ml/min no grupo A e 40 ml/min no Grupo B (*P*<0,001). Valores do índice de pulsatilidade da artéria mamária interna esquerda para o Grupo A e do Grupo B foram de 2,6 e 3,0, respectivamente (*P*<0,001). Não houve diferença estatisticamente significativa entre os parâmetros do enxerto venoso.

**Conclusão:**

Sugerimos um efeito da infusão de diltiazem em aumentar os fluxos de enxerto em operações de bypass de artéria coronária.

**Table:** 

**Abbreviations, acronyms & symbols**	
ACT	Activated coagulation time
COPD	Chronic Obstructive Pulmonary Disease
CPB	Coronary artery bypass graf
CVICU	Cardiovascular intensive care unit
DF	Diastolic filling
IMA	Internal mammary artery
KCl	Potassium chloride
LAD	Left anterior descending artery
LIMA	Left internal mammary artery
MI	Myocardial infarction
NTG	Nitroglycerin
PI	Pulsatility index
Qmean	Mean graft flow
RA	Radial artery
SVG	Vena saphenous vein graft
TTF	Transit-time flowmetry
TTFM	Transit time flow meter

## INTRODUCTION

Owing to technological developments and novel techniques, coronary artery bypass graft (CABG) surgery is successfully performed in many centers.

The human internal mammary artery (IMA) is commonly used as a coronary graft in CABG surgery due to its superior graft patency and increased long-term survival^[1]^.

Graft spasm of the IMA is a long recognized problem, and vasospasm that may develop in arterial grafts during or after the surgery plays an important role in early postoperative mortality and morbidity through impaired myocardial contraction and low output^[2]^.

Despite extensive investigations on the antispastic therapy in the past decades, IMA spasm sometimes still occurs even in the current practice. The mechanism of graft vasospasm is still unclear and many mechanisms can trigger intraoperative and postoperative vasospasm; factors including surgical stimulus, techniques used to remove the graft, or use of distal IMA segment as well as the effect of several mediators released into the circulation due to ischemic reperfusion damage that may result from the removal of cross clamp during cardio-pulmonary bypass (CPB) are involved in the development of vasospasm. Furthermore, reversal and prevention of graft vasospasm are often challenging and the most effective therapy to overcome spasm is still controversial.

Intraoperative transit time flow meter (TTFM) has been widely used to evaluate graft patency in CABG^[3]^. Compared with other methods for graft blood flow measurement, intraoperative TTFM is non-invasive, easy to use, reproducible and provides real-time information about the haemodynamic characteristics of constructed grafts^[4]^. The TTFM can be used to assess early graft function and to predict graft error in CABG, thus allowing for prompt revision of anastomotic imperfection and prevent graft failure^[5]^.

Diltiazem is a benzothiazepine Ca channel blocker that can be used alone or in combination for the treatment of hypertension (HT), angina and Prinzmetal angina and acts by blocking Ca afflux in myocardium and smooth muscle cells of vessels. It prevents extracellular Ca afflux between target cells, decreases intracellular calcium and produces dilatation in systemic arteries^[6]^.

In our study, we aimed to measure the effect of diltiazem in intraoperative graft flows using a quantitative method [transit-time flowmetry (TTF) technique], and to assess the predictive value of measured graft flows on in-hospital postoperative outcomes.

## METHODS

Patients with coronary artery disease who underwent surgery in our clinic between March and July 2013 were included in this prospective, randomized study. This study was approved by the Ethics Committee before initiation. Consent was obtained from the subjects for participation in the study in line with World Medical Association Declaration of Helsinki (World Medicine Assembly, 2004 Tokyo, Japan). Coronary artery bypass graft surgery was performed in the patients in accordance with the coronary artery bypass surgical revascularization indications established by ACC/AHA.

In this study, 140 patients underwent isolated coronary surgery with a total of 361 grafts. All the grafts that are operated were measured by TTFM intra-operatively, among which 205 were arterial and 156 were venous. Age range was 36-81 (mean=59.8). 109 (78%) of the patients were male and 31 (22%) were female. Flow waves, mean graft flow (Qmean) and Pulsatility index (PI) were used to consider graft as patent. Poor ventricular function (ejection fraction ≤ 40%), resting sinusal bradycardia (<55 beat/min), left bundle branch block were the preoperative exclusion criteria. Hemodynamically unstable patients who required infusion of study drugs beyond the ranges of our study protocol, off-pump CABG, valve and additional aortic and non-cardiac surgery were also excluded intra-operatively. Risk classification was performed using the EuroSCORE scale^[7]^ created for surgical risk assessment. Demographic data were evaluated including age, EuroSCORE scale, gender, hypertension, smoking, ejection fraction, diabetes mellitus, the use of intra-aortic balloon pump, peripheral arterial disease, chronic renal failure, chronic obstructive pulmonary disease, previous myocardial infarction (MI), and emergency surgery.

The subjects were divided into two groups; Group A (n=70) consisting of patients who received diltiazem infusion following anesthesia induction and intubation, and Group B (n=70) consisting of patients who didn't receive diltiazem infusion. Diltiazem was given at a dose of 2.5 microgram/kg/min (Diltizem-L, Mustafa Nevzat Pharmaceuticals, İstanbul, Turkey).

### Surgical Procedure

The left IMA (LIMA) was dissected following median sternotomy. Distal IMA was divided under bleeding control before musculophrenic and superior epigastric branches, and prepared as graft with external administration of papaverine.

Radial artery (RA) and right vena saphenous vein graft (SVG) were prepared from non-dominant limbs in eligible patients simultaneously with IMA dissection. The possibility of postoperative extremity ischemia was eliminated using modified Allen test before RA dissection. A three-hundred unit/kg heparin was intravenously administered to maintain activated coagulation time (ACT) over 400 seconds. When this level was achieved, aorta and right atrium were cannulated. The patients were cooled down to 30-32ºC and cross clamp was placed. Extracorporeal circulation was established by roller pump (Sarns 9000 perfusion systems, Ann Arbor, Michigan USA) and membrane oxygenator (Sechrist 3500 HL, Anaheim USA). Pump prime solution was prepared using 1500 ml of Ringer Lactate, 150 ml of 20% mannitol and 60 ml of sodium bicarbonate. Keeping the mean arterial pressure at 50-60 mmHg, pump flow was adjusted at 2.2-2.4 l/m^2^/min. Perfusion was continued in both groups so that hematocrit value would be over 25%.

A three-step cardioplegia was performed to protect myocardium. Following the cross clamp placement, 500 ml of normothermic blood cardioplegia was administered. The Ringer Lactate/blood content of normothermic blood cardioplegia was adjusted as 1:4. Then, topical cooling was administered with Ringer's lactate solution at 4ºC while 10 ml/kg cold crystalloid cardioplegia (St.Thomas sol.) was given at a mean pressure of 40 mmHg. The final cardioplegia was given as a 500 ml of hot shot blood cardioplegia immediately before the cross clamp removal. Distal coronary anastomoses were performed with 7/0 propylene material and proximal anastomoses with 6/0 propylene using continues suture technique. Proximal radial artery and saphenous vein anastomosis were done to aorta. Cross clamp was removed after distal anastomoses were finished and proximal anastomoses were performed under side clamp practice. No hemofiltration was used. Bleeding was controlled at the surgery area after heparin effect was antagonized by protamine. Measurements were performed from the distal part of the graft and before sternum closure and recorded while taking into consideration Qmean, PI and diastolic filling (DF). The patients were taken to cardiovascular surgery intensive care unit after surgery. They were connected to ventilation and DII standard electrocardiogram, arterial pulse oximetry, arterial blood pressure and central venous pressure were monitored.

### Transit time flow measurement

Flow measurement was performed using a transit time flow meter (MediStim VeriQ System, Norway) after all anastomoses were completed and haemodynamic parameters, such as blood pressure and heart rate, became stable. Based on graft diameter, either 3 or 4 mm, a flow probe was used for flow measurement. Measurements were performed from the distal part of the graft and before sternum closure, and flow parameters recorded included Q mean, PI and diastolic filling (DF). Mean arterial pressure was strictly adjusted at 70-90 mmHg during measurements. Gel may be used during short segment IMA measurements to decrease the interposition between the vessel and the tissue and to increase probe-vessel relation. Before the measurements, blood flow was increased by restoring the normal position of the heart by removing pericardial suspenders.

### Evaluation criteria for satisfactory anastomosis

Anastomotic patency was considered satisfactory when the following criteria were met: (i) waveform of blood flow is normal and satisfactory. Blood filling is diastolic dominant. A DF value is >50 %, which is consistent with the characteristics of coronary artery blood flow. (ii) PI is acceptable (<5)^[8]^. Failure to meet either of the criteria indicated an anastomotic defect and required an immediate revision.

All postoperative cardiac surgery patients were taken to a cardiovascular intensive care unit (CVICU). Patients discharged from the CVICU were transferred to a general care ward under the care of the same team. All patients were monitored continuously for a minimum of 24 h.

Postoperative parameters for investigation were as follows: need for revision, prolonged intubation, atrial fibrillation (AF), postoperative early myocardial infarction, postoperative development of acute renal failure and need for hemodialysis, neurological complications, time spent in intensive care and in-hospital mortality.

### Statistics

Data was given as mean ± standard deviation. The significance of demographic and clinical statistical difference between groups was evaluated using Student T test, Mann Whitney U test and Chi square tests. *P* values lower than 0.05 were considered significant. No significant difference was found between the groups in terms of demographic characteristics.

## RESULTS

The study consisted of 140 patients underwent coronary surgery with a total of 361 grafts. All the grafts that are operated were measured by TTF intraoperatively, among which 205 were arterial and 156 were venous. Age range was 36-81 (mean=59.8). 109 (78%) of the patients were male and 31 (22%) were female.

In Group A, infusion with diltiazem was started in this group of patients following anesthesia induction and intubation. In Group A, a total of 202 grafts were performed in 70 patients [69 (34%) LIMA, 63 (31%) saphenous vein, 70 (34%) radial artery], 43 (61%) of the patients were male and 27 (39%) were female. A total of 70 patients, 66 (94%) male and 4 (6%) female were evaluated. Age range was 36-78 with an average of 57.

In Group B, a total of 159 grafts were performed in 70 patients [66 (42%) LIMA, 93 (58%) saphenous vein, 43 (61%) of the patients were male and 27 (39%) were female. Age range was 53-81 with an average of 64.7]. The mean cardiopulmonary bypass time for Group A was 121.8±20.5 and 125±24.3 for Group B (minute, *P*=0.32).

Comparison of the basal charecterictics, preoperative echocardiograﬁc and clinical parameters among patients for Group A and Group B are shown in Table 1. Number of anastomoses, Q mean, and PI and DF values for Group A and Group B patients are shown in Table 2.

**Table 1:** Comparison of the basal charecterictics, preoperative echocardiografic and clinical parameters among patients from Group A and Group B.

Preoperative Parameters	Group A	Group B	*P*
Age	57	64.7	*P*>0.05
EuroSCORE	1.5	2.7	*P*>0.05
Gender	K: %6 (4)	K: %38 (27)	*P*<0.001
	E: %94(66)	E: %62 (43)	
Hypertension	%60 (42)	%56 (39)	*P*>0.05
Smoking	%70 (49)	%53 (37)	*P*<0.037
Diabetes Mellitus	%37 (26)	%31 (22)	*P*>0.05
Ejection Fraction (EF)	58	59	*P*>0.05
Intraaortic Balloon Pump (IABP)	3	8	*P*>0.05
Peripheral Artery Disease	%4 (3)	%10 (7)	*P*>0.05
Chronic Renal Failure	%2 (1)	%10 (7)	*P*>0.05
Chronic Obstructive Pulmonary Disease	%19 (13)	%24 (17)	*P*>0.05
Myocardial Infarction	%25 (18)	%35 (25)	*P*>0.05
Urgent Operation	%2 (1)	%4 (3)	*P*>0.05

**Table 2:** Number, mean graft flow, diastolic filling, and pulsatile index values for Group A and Group B patients.

Group A	Number	Qmean	PI	DF
LIMA-LAD	69	53	2.6	69
RA-OM1	60	50	2.3	65
RA-RCA	10	52	2.0	64
SVG-LAD	1	63	1.5	69
SVG-DIAG	10	34	2.1	71
SVG-OM1	11	44	2.7	62
SVG-RCA	41	52	2.7	63
Group B	Number	Qmean	PI	DF
LIMA-LAD	69	40	3.0	66
SVG-LAD	1	8	4.5	55
SVG-DIAG	11	39	2.4	71
SVG-OM1	43	45	3.3	66
SVG-RCA	38	65	2.8	61

LIMA=left Internal Mammary Artery; LAD=left anterior descending artery; RA=radial artery; OM=obtuse marginal; SVG=saphenous vein graft; DIAG=diagonal artery; RCA=right coronary artery; Q mean=mean graft flow; DF=diastolic filling; PI=pulsatile index

In both Group A and B, LIMA-left anterior descending artery (LAD) anostomosis was performed in 69 patients, while LAD anastomosis was performed in one patient by using SVG because of the inconvenience of LIMA.

The detailed data revealed that the majority of the patients were male in Group A and were statistically different (*P*<0.001).

Smoking was statistically significantly more common in Group A compared to Group B.

In a comparison between Group A and B, no difference was seen with respect to age, EuroSCORE, hypertension, diabetes mellitus, ejection fraction, peripheral arterial disease, chronic renal failure, Chronic Obstructive Pulmonary Disease (COPD), previous MI and emergency surgery.

All the arterial and venous grafts in both groups were recorded by measuring with TTF, and compared for Qmean, PI and DF values. Qmean and PI values were compared for IMA anastomoses, and a significant increase was found in Group A (*P*<0.001) (Table 3).

**Table 3:** The Qmean and PI values compared for IMA anastomoses in Group A and B.

Grafts	Group A (n=69)	Group B (n=66)	*P* value
LIMA - LAD (Q mean)	53 ml/min	40 ml/min	<0.001
LIMA - LAD (P)	2.6	3	<0.001

LIMA=left internal mammary artery; LAD=left anterior descending artery; Q mean=mean graft flow; PI=pulsatile index

No significant difference was observed in the flow measurements in venous graft anastomoses.

Among postoperative follow-up parameters, only the development of atrial fibrillation was significantly different in Group A (6 patients) compared to Group B (16 patients) (*P*<0.020). Other parameters did not show any difference (Table 4).

**Table 4:** Comparison of number of Group A and Group B patients according to postoperative parameters.

	Group A	Group B	*P* value
Need for Revision	4	4	>0.05
Prolonged Intubation	6	10	>0.05
Atrial Fibrillation	6	16	<0.020
Postop MI	0	0	>0.05
Postop HD	1	7	>0.05
Peripheral Embolus	0	0	>0.05
Neurologic Complication	3	2	>0.05
Duration of Intensive Care	4	3.3	>0.05
Mortality	0	4	>0.05

MI=myocardial infarction; HD=hemodialysis; Postop=postoperative

## DISCUSSION

In this study, by using intraoperative TTFM, it was observed a statistically significant increase in arterial graft flows intraoperatively, and a lower incidence of atrial fibrillation during intra- and postoperative periods in the group that was given diltiazem.

Coronary artery spasm and the arterial graft that occurs during and early after coronary artery surgery can result in a sudden and severe cardiopulmonary failure. The mechanisms underlying intraoperative and postoperative vasospasm are not clearly defined. Many theories have been proposed including techniques used to remove the arterial graft, inappropriate manipulations for the graft, over activation of Ca channels, increase in alpha-adrenergic activity, high blood pH, low body temperature, increased vasopressin levels, excessive release of histamine, low PaCO_2_, use of distal 1/3 part of IMA for anostomosis and smoking^[9,10]^. In addition, accumulation of depolarizing agents as KCl (potassium chloride), free radicals induced by alpha-adrenergic receptors, and increase in agents as arginine or vasopressine also play role in vasospasm^[11]^.

In recent years, arterial grafts other than IMA have become prominent since long term occlusion rates are high with saphenous vein. The most commonly used is the RA. Despite their high rate of staying patent, the most important problem when using arterial grafts is their susceptibility to vasospasm during perioperative period. In a meta-analysis issued by Wijeysundera & Beattie^[12]^, it was demonstrated that intraoperative use of diltiazem reduces the incidence of ischemia and supraventricular tachycardia, while being useful in reducing death and myocardial infarction in postoperative period.

Since vasospasm is induced by various pathways, generation of a synergic effect through simultaneous inhibition of different pathways by using Ca channel blockers and Nitroglycerin (NTG) concomitantly has been studied. Chanda et al.^[13]^ reported that concomitant use of diltiazem and NTG results in better outcomes in loosening of the contraction compared to when each drug is used alone.

Lemmer et al. reported in their study that intracoronary infusion of nitroglycerin had a beneficial effect in the loosening of vasospasm when used together with calcium channel blockers and that Ca channel blockers would have a beneficial effect on ventricular dysfunction^[14]^.

In the study of Tabel et al.^[15]^, half of the patients undergoing elective CABG were given diltiazem and the other half was given NTG together with anesthesia induction. Two measurements had been taken. First, free flow value was measured after IMA was cut from bifurcation level. Then, the second flow measurement was performed after the distal segment was resected. The flow values were found to be 53.8 ml/min in the first measurement and 72.3 ml/min in the second in diltiazem group while the same values were 25.7 ml/min and 48.9 ml/min, respectively, in NTG group (*P*=0.000, 0.004, respectively). After the resection of distal segment, both agents increased the flow (*P*=0.000, 0.000). However, they reported that diltiazem was more effective in preventing IMA spasm. In our study, the flow value in LIMA-LAD graft was 53 ml/min in the group that were given diltiazem and 40 ml/min in the group without diltiazem (*P*<0.001). Inokuchi et al.^[16]^ reported that fasudil, a Rho-kinase inhibitor, is effective in suppressing coronary artery spasm in patients with vasospastic angina.

The IMA flow can be increased by administering vasodilators such as Ca channel blockers, long acting nitrates and phosphodiesterase inhibitors during surgery. Otherwise, this condition is associated with high mortality and morbidity. However, these agents are rather effective in decreasing graft spasm than their vasodilator effect. Apart from its effect on vasospasm, we discovered in our study that intraoperative use of diltiazem also reduces the development of atrial fibrillation. There was a statistically significant decrease compared to the other group (*P*<0.02). Besides its effect on arrhythmia, ventricle protecting and anti-ischemic effects have also been reported in the literature.

In a double blind, randomized study of Zhang et al.^[17]^ on 71 elective CABG patients, diltiazem infusion provided a better protection against both ischemia and supraventricular arrhythmia.

It was also reported that preoperative diltiazem was effective in reducing arrhythmia and transient ischemic events in addition to prevention of IMA spasm^[18]^.

The predicted blood flow through LIMA-LAD graft is 25-50 ml/min^[19]^. This value is higher in venous grafts. That is because IMA cannot get adapted to the sudden decrease in the peripheral vascular resistance and increasing outflow. However, graft measurements in one year have shown a doubled amount in blood flow.

In the past, a number of methods had also been used to measure graft patency. Much better results have been reported with intraoperative TTF^[20]^. Perioperative flow measurement protects the patient from unjustifiable complications and provides the surgeon with patency of the graft. We consider that use of TTF in CABG is important in order to achieve a high success and low mortality/morbidity. TTF measurement is fast, economic, effective and is a simple alternative that helps the surgeon compared to methods as CAG. Abnormal flow was seen in five of the grafts and revision was performed. We considered a possible anastomosis error in five patients because of various reasons when all three parameters were assessed. All of these parameters recovered when the anostomoses were fixed. Thus, we protected the patients from any postoperative complications.

The present study has several limitations. First of all, this study is not "surgeon-blind", and the control group did not fully reflect features of the study group. The number of patients who were included in the study was another limitation of our study. Although we adjusted for many clinical and procedural characteristics, hidden confounders cannot be excluded, we did not measure other markers of specific oxidative stress and inflammation which may be associated with vasospasm, such as free radical levels. In the studies, long term benefits were demonstrated for arterial grafts used in coronary revascularization. Other studies should be performed in the future in order to understand the reasons and prevent arterial graft spasm which is the most important problem during intraoperative and early preoperative period. In these studies, the novel techniques and treatment modes developed to prevent vasospasm will contribute to the reduction of mortality and morbidity of future CABG surgery.

## CONCLUSION

In conclusion, the beneficial effect of diltiazem on LIMA flows in patients undergoing CABG surgery was found in this study, we therefore recommend diltiazem infusion in increasing LIMA graft flows.

**Table:** 

**Authors’ roles & responsibilities**	
OE	Analysis and/or interpretation of data; final manuscript approval; implementation of projects and/or experiments
MEM	Analysis and/or interpretation of data; final manuscript approval; implementation of projects and/or experiments
AIT	Analysis and/or interpretation of data; study design; implementation of projects and/or experiments
UA	Conduct of operations and/or experiments
OK	Analysis and/or interpretation of data; implementation of projects and/or experiments
RK	Analysis and/or interpretation of data
IG	Study conception and design
